# Supportive Text Messaging and Peer Support for Patients in the 6 Months Following Discharge from a Psychiatric Admission: Mental Health Outcomes from a Cluster-Randomized Controlled Trial

**DOI:** 10.3390/jcm14238262

**Published:** 2025-11-21

**Authors:** Wanying Mao, Reham Shalaby, Ernest Owusu, Hossam Eldin Elgendy, Belinda Agyapong, Peter H. Silverstone, Xin-Min Li, Andrew J. Greenshaw, Ejemai Eboreime, Wesley Vuong, Arto Ohinmaa, Vincent I. O. Agyapong

**Affiliations:** 1 Department of Psychiatry, University of Alberta, Edmonton, AB T6G 2H5, Canada; wmao2@ualberta.ca (W.M.); rshalaby@ualberta.ca (R.S.); eowusu2@ualberta.ca (E.O.); hossamel@ualberta.ca (H.E.E.); bagyapon@ualberta.ca (B.A.); peter.silverstone@me.com (P.H.S.); xinmin@ualberta.ca (X.-M.L.); agreensh@ualberta.ca (A.J.G.); 2 Department of Psychiatry, Dalhousie University, Halifax, NS B3H4K3, Canada; ejemai.eboreime@dal.ca; 3 Alberta Health Services, Addiction and Mental Health Services, Edmonton, AB T5J 0K1, Canada; wesley.vuong@albertahealthservices.ca; 4 School of Public Health, University of Alberta, Edmonton, AB T6G1C9, Canada; arto.ohinmaa@ualberta.ca

**Keywords:** anxiety, depression, well-being, psychiatric discharge, supportive interventions, community care

## Abstract

**Background/Objective:** The transition from psychiatric inpatient care to community settings poses risks of relapse, rehospitalization, and poor well-being. This study examined changes in anxiety, depression, suicidal ideation, sleep issues, and well-being over six months post-discharge and assessed the effectiveness of supportive text messaging (Text4Support) alone and with peer support, compared to treatment as usual (TAU). **Methods:** A pragmatic stepped-wedge cluster-randomized trial included 1098 participants discharged from psychiatric units across Alberta, Canada. Participants were allocated to the TAU, Supportive Text Messaging (SMS), or SMS plus peer support (SMS+PS) group. Outcomes were measured using GAD-7, PHQ-9, and WHO-5 at baseline and at six weeks, three months, and six months post-discharge. ANCOVA compared outcomes across groups at each time point, controlling for baseline values. **Results:** Follow-up completion declined (20% at six weeks, 16% at three months, and 15% at six months). No group differences emerged for anxiety, depression, suicidal ideation, or sleep issues at six weeks or three months. Well-being was significantly higher in the SMS group at six weeks (η^2^ = 0.10). At six months, between-group differences appeared for anxiety and depression, though post hoc tests showed no pairwise differences. **Conclusions:** Supportive text messaging may improve well-being shortly after discharge and holds promise as a low-intensity transitional care strategy, though findings are limited by low follow-up.

## 1. Introduction

The transition from psychiatric inpatient care to community-based services represents a critical phase in the recovery process for individuals experiencing mental health conditions. This period is often accompanied by heightened risks of relapse, readmission, and increased mortality [1,2]. Previous research consistently shows that patients discharged from psychiatric facilities face substantial challenges and adverse outcomes, particularly within the initial months following discharge [1,2,3]. For example, a study published in *The British Journal of Psychiatry* highlighted that approximately 13% of psychiatric patients are readmitted shortly after discharge, reflecting their vulnerability during the transition to outpatient care [4].

Similar trends are observed within the Canadian context. According to data from the Canadian Institute for Health Information (CIHI), mental health and substance use disorders significantly contribute to hospital readmissions within 30 days post-discharge [5]. Specifically, CIHI reports indicate that one in nine mental health inpatient discharges leads to readmission within 30 days [5]. These statistics underscore systemic shortcomings in the provision of adequate post-discharge support and accessible community-based services. Factors contributing to high readmission rates and adverse outcomes post-discharge include unmet mental healthcare needs and limited community mental health resources. Indeed, evidence from BioMed Central (BMC) Health Services Research demonstrates that individuals discharged from inpatient psychiatric units exhibit some of the highest readmission rates across hospital populations, often driven by inadequate follow-up and community support [6].

In response to these challenges, various transitional interventions have been developed and implemented internationally [1,7,8]. These interventions typically aim to offer continuous support during the critical post-discharge period, reducing the likelihood of readmission and promoting sustained recovery. Some interventions specifically address the needs of vulnerable groups, such as individuals at risk of homelessness post-discharge [8,9]. Others, including medication management programs, focus on mitigating particular health risks associated with the transition period [10,11]. Additionally, broader interventions seek to enhance coordination among different healthcare and support services, ensuring a seamless transition [8,12]. A systematic review published in The British Journal of Psychiatry found that structured discharge planning and transitional support services effectively reduce early psychiatric readmissions [4]. In Alberta, Canada, innovative approaches have been introduced to bridge gaps between inpatient and outpatient care. Notably, the Text4Support program, which provides daily supportive text messages, as well as peer support initiatives, have been designed to address gaps in community-level support for individuals transitioning from acute care to community mental health services [6]. These initiatives aim to reduce psychological distress and bridge the treatment gap faced by patients awaiting further psychiatric services from Alberta Mental Health.

The current study investigates the progression of mental health symptoms over six months following discharge from psychiatric hospitalization. Specifically, it compares the effectiveness of the Text4Support program and a peer support intervention against treatment as usual (TAU). Employing validated instruments, including the Generalized Anxiety Disorder 7-item scale (GAD-7), the Patient Health Questionnaire-9 (PHQ-9), and the World Health Organization-Five Well-Being Index (WHO-5), this research aims to assess changes in anxiety, depression, suicidal ideation, sleep disturbances, and overall well-being among participants. Additionally, the study seeks to identify factors influencing mental health outcomes during the post-discharge period, providing critical insights for refining future interventions and enhancing community support strategies.

This study is conceptually informed by the biopsychosocial model and social support theory, which together explain how supportive communication and interpersonal connection can promote recovery after psychiatric discharge. Consistent with these frameworks, supportive text messaging is designed to enhance perceived social connectedness, coping self-efficacy, and emotional regulation through brief, repeated affirmations. Similarly, peer support provides experiential validation and models recovery-oriented behaviors that strengthen patients’ sense of belonging and empowerment during the vulnerable post-discharge period.

## 2. Materials and Methods

### 2.1. Study Setting and Design

This study employed a pragmatic stepped-wedge cluster-randomized design and was conducted across ten major healthcare sites in Canada, Alberta province, including Edmonton, Calgary, and Grand Prairie, from 8 March 2022 to 29 February 2024. The study aimed to evaluate the effectiveness of post-discharge interventions, namely supportive text messaging (Text4Support) alone or combined with peer support, compared TAU.

While participants in the TAU and SMS groups followed the randomized rollout schedule, the SMS+PS group included patients identified by clinical teams as being at higher risk of readmission. This design reflects real-world implementation where additional support is prioritized for clinically complex patients.

The Text4Support program delivered one supportive text message daily for six months after discharge. Messages addressed themes such as coping with stress, emotional regulation, self-care, and help-seeking, and included diagnosis-specific content tailored across six psychiatric categories: mood, anxiety, psychotic, substance use, adjustment, and personality disorders. Messages were standardized and not dynamically adjusted post-enrollment. Example Text4Support messages included: “Take one step at a time, progress, not perfection, leads to recovery,” “Reach out when you feel overwhelmed; connection is strength,” and “Remember to rest and care for yourself today, you matter.”

The Peer Support component involved trained peer support workers with lived experience of mental illness and recovery. They offered weekly in-person or phone sessions focusing on emotional validation, problem-solving, relapse prevention, and connection to community resources. Peer workers followed a structured recovery framework emphasizing empowerment, hope, and self-determination, and participated in regular clinical supervision to ensure fidelity.

These interventions were designed to address gaps in community-based support and service continuity, that is, the limited accessibility of outpatient resources, fragmented coordination between hospital and community services, and reduced social connectedness experienced by many discharged patients.

### 2.2. Data Collection and Inclusion Criteria

A face-to-face recruitment process was conducted from 8 March 2022 to 29 February 2024 across ten major sites in Edmonton, Calgary, and Grand Prairie, Alberta, Canada. Nurse managers and staff supported the research team by identifying potential participants who were nearing discharge from inpatient psychiatric units in the designated hospitals. Eligible patients were provided with comprehensive information about the study, including an information leaflet. Patients who expressed interest signed a paper-based consent form and completed a self-administered questionnaire using a tablet device. The survey was developed on REDCap, a secure browser-based platform designed for creating and managing online surveys and research databases, with support from research team members [13]. Data collected included socio-demographic details (e.g., age, gender, ethnicity, relationship status, employment status, education level, housing status) and clinical characteristics (e.g., mental health diagnosis at discharge, levels of anxiety, depression, and well-being). All enrolled participants were included in the analysis.

Inclusion and Exclusion Criteria: Participants were required to have a diagnosed mental health condition, be preparing for discharge from an inpatient psychiatry unit, aged 18 years or older, and possess a mobile device with an active phone number. They were also required to have the ability to receive and read text messages in English and provide written informed consent. Patients planning to travel out of town during the six-month follow-up period were excluded due to the nature of one of the interventions, a peer support service, which necessitated in-person meetings to provide lived experiences shared by peer support workers. The research team collected participants’ phone numbers and healthcare numbers, which served as primary identifiers.

### 2.3. Ethics Statement

Ethical approval for this study was obtained from the University of Alberta’s Health Research Ethics Board (Ref # Pro00111459). The regional health authority also provided additional operational approval. Written informed consent was obtained from all participants.

### 2.4. Sample Size Calculation

The sample size was calculated based on a population of 28,571 psychiatric discharges in Alberta in 2018. Using a 95% confidence interval and a ±3% margin of error, the required minimum sample size was estimated at 1036 participants after rounded up slightly for a conservative estimate using an online script [14]. This ensures adequate statistical power to detect meaningful differences across groups while maintaining representativeness and generalizability to the broader psychiatric inpatient population.

### 2.5. Outcome Measures

#### 2.5.1. Generalized Anxiety Disorder Scale (GAD-7)

The Generalized Anxiety Disorder 7-item scale (GAD-7) was utilized to assess anxiety symptoms among participants. This self-report instrument comprises seven items designed to evaluate the frequency and severity of anxiety symptoms experienced over the past two weeks. Respondents rate each item using a 4-point Likert scale ranging from 0 (“Not at all”) to 3 (“Nearly every day”), yielding a total possible score ranging from 0 to 21. Higher scores correspond to greater severity of anxiety symptoms [15]. The GAD-7 is widely used in both clinical practice and research due to its strong psychometric properties. At the recommended cut-off score of 10, the GAD-7 exhibits a sensitivity of 89% and a specificity of 82% for identifying cases of generalized anxiety disorder. Additionally, the measure demonstrates strong test–retest reliability (intraclass correlation coefficient = 0.83) and excellent internal consistency (Cronbach’s α = 0.92) [15,16].

#### 2.5.2. Patient Health Questionnaire (PHQ-9)

The Patient Health Questionnaire-9 (PHQ-9) was employed to measure depressive symptoms among participants. Based on the nine diagnostic criteria for major depressive disorder outlined in DSM-IV, this self-administered questionnaire captures the frequency of depressive symptoms experienced over the preceding two weeks. Each item is rated on a scale from 0 (“Never”) to 3 (“Nearly every day”), resulting in total scores ranging from 0 to 27 [17,18]. The PHQ-9 serves both as a diagnostic tool for major depressive disorder and as an indicator of symptom severity. A threshold score of 10 or higher is generally recommended to identify significant depression. Severity levels are classified as mild (5), moderate (10), moderately severe (15), and severe (20). The instrument demonstrates good internal consistency (Cronbach’s α = 0.85) and strong convergent validity through moderate-to-high correlations with measures such as WHO-5, HADS-depression, HADS-anxiety, and GAD-7. The PHQ-9 displays high sensitivity and specificity at approximately 88% and a cut-off score of ≥10 [17,18,19].

Item 3 of the PHQ-9 (PHQ sleep) [20,21] and item 9 (suicide ideation) [22] are specific indicators within the PHQ-9 scale used to assess sleep disturbances and suicidal risk, respectively. PHQ sleep explicitly asks respondents about difficulties falling asleep, staying asleep, or sleeping excessively over the previous two weeks. Evidence from primary care research confirms its utility and efficiency as a reliable screening tool for sleep problems without imposing a significant respondent burden [20,21,23]. For example, MacGregor et al.’s study in a Veterans Affairs Primary Care setting demonstrated that PHQ sleep scores correlated strongly with established measures like the Insomnia Severity Index, with a score of 1 effectively balancing sensitivity and specificity [23]. Similarly, PHQ-9 item 9, assessing suicidal ideation (“Thoughts that you would be better off dead or hurting yourself”), serves as a significant predictor of suicidal risk, independent of overall depression severity [22]. Research indicates that higher scores on this item substantially predict suicide risk and mortality, with reported suicidal ideation on this item robustly linked to suicide attempts and deaths for up to two years [24]. Specifically, the PHQ-9 suicide item exhibited a sensitivity of 0.69 and specificity of 0.84, underscoring its utility for routinely identifying suicide risk in primary care settings, thus facilitating intervention for individuals who might otherwise remain undetected [25].

#### 2.5.3. World Health Organization-Five Well-Being Index (WHO-5)

The World Health Organization-Five Well-Being Index (WHO-5) was administered to assess overall mental well-being among participants. Developed by the World Health Organization, this brief self-report scale includes five positively phrased statements assessing subjective well-being, such as “I have felt cheerful and in good spirits,” “I have felt calm and relaxed,” and “My daily life has been filled with things that interest me” [26]. Respondents rate each item on a 6-point Likert scale, ranging from 0 (“Not at all”) to 5 (“All the time”), with scores converted to a standardized scale from 0 (lowest well-being) to 100 (highest well-being). Scores below 50 indicate poor emotional well-being and warrant further clinical evaluation. The WHO-5 demonstrates strong internal consistency reliability (Cronbach’s α = 0.90), good convergent validity with measures such as the PHQ-9 (r = −0.73, *p* < 0.001), and high sensitivity (93%) and specificity (83%) in identifying depression [26,27].

### 2.6. Statistical Analysis

All statistical analyses were conducted using SPSS Version 25 [28]. Descriptive statistics were employed to summarize participants’ sociodemographic and clinical characteristics against the study arms (TAU, SMS, SMS+PS), presented as frequencies and percentages. To evaluate the changes in anxiety (GAD-7), depression (PHQ-9), suicidal ideation, sleep disturbances, and well-being (WHO-5) across the three study groups (TAU, SMS, SMS+PS) at each follow-up time point (six weeks, three months, six months), analysis of covariance (ANCOVA) was utilized, controlling for baseline scores of the respective scales. Assumptions of ANCOVA, including homogeneity of regression slopes, were assessed and satisfied, with outcome measures (GAD-7, PHQ-9, and WHO-5 scores at 6 weeks, 3 months, and 6 months post-discharge) serving as the dependent variables, and study arms (Treatment as Usual, SMS, and SMS plus Peer Support) as the independent variable.

## 3. Results

### 3.1. Summary of Study Recruitment and Enrollment

**Figure 1:**
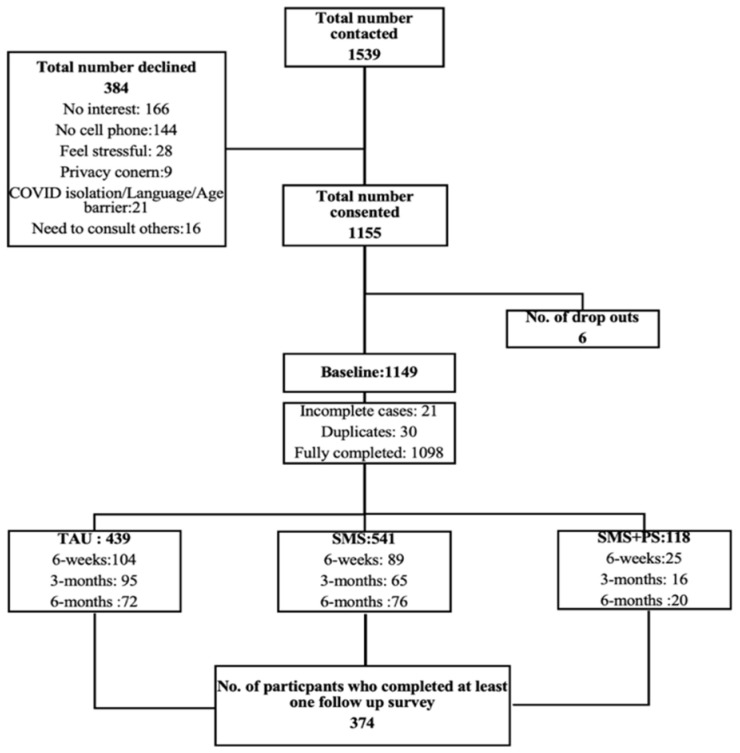
Flowchart of participant recruitment and enrollment process.

Figure 1 illustrates the flowchart of participant recruitment and enrollment for the current study. Initially, the research team approached a total of 1539 patients who had undergone assessment and fulfilled the predefined inclusion criteria for participation. Among these eligible individuals, 384 patients opted not to participate in the study, providing various reasons such as lack of interest in research, absence of a cell phone, or concerns regarding potential stress associated with participation. Consequently, 1155 patients consented to partake in the study. Following consent, six participants subsequently decided to withdraw from the study with no disclosed reasons, resulting in 1149 participants at baseline. During the subsequent data cleaning phase, the research team identified and excluded 21 incomplete responses and 30 duplicate entries, resulting in a final sample size of 1098 fully completed and valid cases for analysis. Utilizing a pragmatic stepped-wedge cluster-randomized design, these participants were distributed into three distinct groups.

### 3.2. Baseline Data Analysis

Demographic and clinical data were collected from the entire sample of participants (n = 1098), with the study arms as a comparator variable (Table 1 and Table 2). The sample included 439 (40%) participants who were assigned to treatment as usual (TAU) group; 541 (49.3%) belonged to text message (SMS) group; 118 (10.7%) respondents belonged to peer support group. A total of 401 (36.5%) of participants were between 18 and 25 years old; 376 (34.2%) of them were between 26–40 years old; and 321 (29.2%) of them were above 40 years old. Among study participants, 486 (42.6%) respondents were male, 598 (54.5%) were female, and 32 (2.9%) were other genders. Most respondents, 675 (61.5%), were Caucasian; 565 (51.5%) had a high school diploma; 647 (58.9%) were single; 585 (52.9%) were unemployed; 458 (41.8%) lived with family or friends, and 290 (26.4%) of the respondents had been diagnosed with depression. Table 1 gives a more detailed description of the characteristics of the respondents.

### 3.3. ANOVA Analysis

Table 2 presents the results of ANOVA (Analysis of Variance) analyses comparing clinical baseline measures across the three intervention groups: TAU, SMS, and SMS+PS. A statistically significant difference was observed in baseline anxiety symptoms, as measured by the Generalized Anxiety Disorder scale (GAD-7) (F (2) = 8.94, *p* = 0.01). The TAU group reported the highest mean anxiety score (M = 8.78, SD = 5.76), followed by the SMS group (M = 8.32, SD = 5.71), while the SMS+PS group reported the lowest mean score (M = 7.77, SD = 4.67). In contrast, ANOVA results indicated no significant differences among the groups at baseline for depression (PHQ-9), well-being (WHO-5), suicidal ideation, or sleep-related problems (all *p*-values > 0.05).

**Table 2:** Clinical baseline measures among the study participants.

Variable	TAU (M ± SD)	SMS (M ± SD)	SMS+PS (M ± SD)	Chi Square	df	*p*-Value
**GAD-7**	8.78 ± 5.76	8.32 ± 5.71	7.77 ± 4.67	8.94	2	0.01 *
**PHQ-9**	11.01 ± 6.56	11.39 ± 6.80	11.26 ± 5.27	1.86	2	0.40
**WHO-5**	51.68 ± 23.38	52.02 ± 23.43	47.80 ± 21.33	3.62	2	0.16
**Suicidal Ideation**	0.76 ± 0.96	0.79 ± 0.99	0.75 ± 0.81	7.51	6	0.75
**Sleep Issues**	1.44 ± 1.06	1.48 ± 1.04	1.49 ± 0.86	3.47	6	0.28

* Significant *p*-value.

### 3.4. ANCOVA Analysis

ANCOVA was conducted to evaluate the effectiveness of treatment as usual, SMS alone, and SMS plus peer support in managing depression, anxiety, and well-being across time points (Table 3). Assumption testing confirmed that the homogeneity of regression slopes was met at all time points (*p* > 0.05). On the GAD-7 scale, after adjusting for baseline scores, there were no statistically significant differences in anxiety scores between the three groups at six weeks (F (2, 221) = 0.12, *p* = 0.89, η^2^ = 0.00) or three months (F (2, 166) = 0.23, *p* = 0.80, η^2^ = 0.00). At six months, a statistically significant difference emerged (F (2, 153) = 2.78, *p* = 0.01, η^2^ = 0.10). However, subsequent pairwise comparisons (Table 4) did not yield any statistically significant differences between groups (all *p* > 0.05), indicating that while a group effect was detected overall, no individual group outperformed another to a statistically significant degree.

Depressive symptoms showed no significant group differences at six weeks (F (2, 226) = 0.91, *p* = 0.40, η^2^ = 0.01) or three months (F (2, 173) = 0.28, *p* = 0.76, η^2^ = 0.00). A statistically significant group difference was observed at six months (F (2, 161) = 2.50, *p* = 0.03, η^2^ = 0.09). Although participants in the SMS group appeared to show the greatest reduction in depression symptoms at six months, the post hoc analysis (Table 4) did not reveal any statistically significant differences between specific groups (all *p* > 0.05). This indicates that, despite observable trends, the variations in mean scores across groups were not substantial enough, after adjusting for multiple comparisons, to attribute a clear advantage to any intervention.

A statistically significant difference in well-being was observed at six weeks (F (2, 227) = 4.24, *p* = 0.00, η^2^ = 0.10), though the difference did not persist at three months (F (2, 161) = 1.89, *p* = 0.09, η^2^ = 0.06) or six months (F (2, 1086) = 0.14, *p* = 0.87, η^2^ = 0.00). Post hoc tests at six weeks (Table 4) found no significant pairwise differences (all *p* > 0.05), suggesting that while group means differed significantly overall, the magnitude of difference between individual groups was not sufficient to be considered statistically significant.

No statistically significant differences were observed among the groups for suicidal ideation at six weeks (F (2, 226) = 1.15, *p* = 0.32, η^2^ = 0.01), three months (F (2, 174) = 0.14, *p* = 0.87, η^2^ = 0.00), or six months (F (2, 161) = 2.00, *p* = 0.07, η^2^ = 0.07). Likewise, no significant differences were found in sleep issues across any time points (six weeks: F (2, 226) = 0.18, *p* = 0.84, η^2^ = 0.00; three months: F (2, 174) = 0.06, *p* = 0.94, η^2^ = 0.00; six months: F (2, 161) = 0.72, *p* = 0.63, η^2^ = 0.03).

## 4. Discussion

This study assessed the effectiveness of supportive text messaging and peer support interventions on mental health outcomes following discharge from psychiatric inpatient care, using a pragmatic stepped-wedge cluster-randomized controlled trial design. The primary aim was to compare the impact of these interventions on anxiety, depression, suicidal ideation, sleep disturbances, and overall well-being over a six-month period post-discharge. Overall, the findings highlight the complexities of sustaining symptom improvements, underscore the challenges of maintaining mental health improvements after discharge and emphasize the importance of structured, ongoing transitional support to address patients’ continuing needs.

Baseline analyses revealed substantial prevalence rates of psychiatric symptoms among participants. Over half met the criteria for likely depression, nearly half reported poor well-being, and a considerable proportion expressed suicidal ideation shortly before discharge. These findings underscore the vulnerable state of psychiatric patients at hospital discharge, reinforcing the literature indicating heightened risks for relapse, readmission, and adverse psychological outcomes following the immediate transition to community care [29,30,31,32]. The demographic profile further highlights common social vulnerabilities, with high rates of unemployment, single status, and relatively young age, factors previously associated with increased psychiatric morbidity and limited coping resources during transitional periods [30,32].

ANCOVA results revealed no statistically significant differences among the three intervention groups at six weeks or three months post-discharge for anxiety, depression, suicidal ideation, or sleep disturbances. However, well-being scores differed significantly at six weeks, with a moderate effect size (η^2^ = 0.10). This suggests that short-term interventions may positively influence subjective well-being, potentially by increasing perceived social support and fostering a sense of connectedness, as supported by earlier studies [32,33].

It is important to note, however, that baseline analysis revealed a statistically significant difference in anxiety symptoms across the groups, with the TAU group reporting higher GAD-7 scores at discharge compared to the SMS and SMS+PS groups. Although this difference was modest, it may have influenced the patterns of anxiety symptom progression observed over time. Consequently, interpretations of group differences, particularly regarding anxiety outcomes, should be made cautiously.

The lack of significant findings across all measured outcomes at three months potentially across indicates more transient nature for the initial intervention effects. While participants may initially benefit from enhanced feelings of support and engagement provided by text messaging or peer interaction, these effects might diminish without sustained, active therapeutic engagement. This raises important questions regarding intervention sustainability and suggests that ongoing support rather than short-term interventions may be essential for required improvements in mental health outcomes post-discharge.

At six months, statistically significant differences emerged among groups for both anxiety and depression scores. However, post hoc analyses revealed that no specific pairwise comparisons reached statistical significance. While the SMS group consistently showed lower anxiety and depression scores at six months compared to the TAU and SMS+PS groups, these trends were not large enough to conclude a definitive treatment advantage. Thus, while the effect sizes suggest some potential clinical relevance (η^2^ = 0.10 for anxiety, η^2^ = 0.09 for depression), results must be interpreted with caution given the absence of statistically significant between-group differences. The findings may indicate delayed or cumulative intervention effects, particularly for low-intensity digital interventions like SMS, but this remains speculative [32,33]. The lack of significant pairwise differences at six months, despite overall ANCOVA significance, likely reflects a combination of modest effect sizes and limited follow-up sample size. This aligns with evidence from previous pragmatic digital intervention trials where statistical power decreased with attrition. It is also plausible that low-intensity interventions such as supportive text messaging require extended or continuous delivery to sustain symptom improvements. The early but short-lived enhancement in well-being observed at six weeks may stem from increased perceived social connectedness; prior research indicates that supportive digital contact can enhance well-being by reinforcing a sense of belonging and emotional validation. At six months, while differences in anxiety and depression scores reached overall significance, post hoc tests showed no pairwise differences, suggesting that the statistical power was insufficient to detect group-level differences despite clinically meaningful trends. Future studies should assess dose–response relationships and determine optimal intervention duration and frequency.

For well-being, although significant differences were observed at six weeks, these were not sustained at three or six months. The early but short-lived improvement in well-being may point to an initial “lift” associated with receiving supportive messages, a pattern observed in other digital mental health interventions [33]. However, the lack of sustained differences highlights the challenge of maintaining gains without continued engagement.

The absence of significant findings regarding suicidal ideation and sleep disturbances across all measurement points merits attention. These symptoms, often indicators of acute psychiatric distress, may be less responsive to general supportive interventions, and require more targeted interventions beyond supportive text messages or peer support. This suggests that future intervention strategies should incorporate more specialized therapeutic approaches, such as cognitive-behavioral therapy for insomnia and crisis intervention for suicidal ideation to address specific symptom domains effectively [32,34].

Notably, the SMS+PS group consisted of individuals who had been clinically assessed as being at higher risk of adverse outcomes following discharge. This selection criterion introduces potential bias, as participants in this group may have had more complex clinical profiles or greater symptom severity at baseline, which could have influenced the intervention outcomes. In addition, participants may have engaged more consistently with the SMS-only program due to its simplicity, accessibility, and lower burden of participation [30,32]. In contrast, the peer support component may have introduced variability in both the delivery and the degree of participant engagement [30,32]. These insights highlight that the effectiveness of peer support interventions may depend heavily on the consistency, quality, and personalization of the peer interactions. From a service delivery perspective, the findings reinforce the need for post-discharge programs that are not only easily accessible and low barrier but also adaptable to individual patient needs. Personalized care pathways that take into account patients’ baseline symptom severity, communication preferences, and available social supports could optimize outcomes [34]. Moreover, integrating scalable interventions such as SMS with periodic reassessments and stepped-care models, where the intensity of support is increased for individuals with persistent or severe symptoms, may enhance the long-term effectiveness of transitional care programs [35].

Importantly, demographic and clinical characteristics at baseline revealed substantial levels of distress and vulnerability among participants. High prevalence rates of depression, anxiety, and suicidal ideation at discharge reflect systemic challenges in adequately addressing these issues within inpatient settings. Post-discharge interventions alone may not suffice unless supported by broader systemic improvements in inpatient and community care continuity. Collaborative care models involving multidisciplinary teams could be critical to enhancing transitional care, ensuring that community services effectively complement discharge planning [33,36].

The study findings have important implications for healthcare providers, policymakers, and community mental health services. Specifically, these results suggest a need to reevaluate and potentially redesign post-discharge support programs [37,38]. The identified need for sustained engagement points towards integrating technology-based interventions with traditional care strategies to enhance continuity and adherence [39,40]. Mental health services could benefit from structured follow-up programs involving periodic mental health assessments and personalized support adjustments, leveraging digital communication channels to maintain regular contact with discharged patients [40]. Our results also highlight the need for personalized care approaches, reflecting varying patient needs over the post-discharge period [41]. Given that not all patients respond uniformly to supportive messaging or peer interactions, tailoring interventions based on individual characteristics (e.g., severity of symptoms, social support availability, preference for communication methods) could optimize outcomes. Personalized intervention plans, possibly informed by initial patient assessments, may enhance effectiveness and patient satisfaction [41].

Moreover, these findings support the argument for greater integration and coordination between inpatient and outpatient mental health services [42,43]. Establishing robust transitional care protocols, including discharge planning, community outreach programs, and enhanced resource allocation, could significantly reduce post-discharge relapse rates and improve overall patient outcomes [42,43]. Cross-sector collaborations involving healthcare, social services, and community organizations might ensure comprehensive, accessible, and continuous care during vulnerable transition periods [43].

Further investigation is required to understand mechanisms underlying engagement and adherence in post-discharge populations. Mixed-methods studies incorporating qualitative interviews and quantitative analyses could provide deeper insights into patient experiences, perceptions of intervention usefulness, and barriers to sustained engagement. Such studies could also explore the potential additive effects of peer support combined with digital interventions, offering comprehensive evidence to inform future service development.

## 5. Limitations

This study has several limitations. The relatively low follow-up completion rate across time points may have introduced attrition bias, limiting representativeness. Due to anonymized data linkage, dropout analyses comparing completers and non-completers could not be conducted. The non-random allocation of participants to the SMS+PS group, which was based on clinical assessment of high readmission risk, may have introduced selection bias. The smaller sample size in the SMS+PS group reflects its targeted inclusion of patients deemed at higher clinical risk, which resulted in unequal group sizes. This may have limited statistical power for between-group comparisons. While baseline symptom scores were controlled for in ANCOVA models, future studies should employ stratified or matched randomization to enhance comparability. Additionally, concurrent treatments and community services received post-discharge were not systematically tracked, which may confound observed effects. Future research should collect such data to isolate the independent impact of text messaging and peer support interventions. Finally, the pragmatic implementation context and provincial setting may limit generalizability to other health systems.

## 6. Conclusions

This study underscores the complexities of supporting psychiatric patients post-discharge, highlighting that while short-term interventions may enhance subjective well-being, sustained interventions could yield more significant clinical improvements in anxiety and depression symptoms over extended periods. To maximize long-term outcomes, future interventions should incorporate personalized, sustained, and integrated care approaches. Addressing barriers to patient engagement and enhancing coordination between inpatient and community mental health services is essential for improving transitional care and reducing the risk of relapse and readmission.

## Figures and Tables

**Table 1:** Distribution of socio-demographic and clinical characteristics among the study participants.

Variables	Treatment Groups			
	TAU (n = 439) n (%) = 40.0%	SMS (n = 541) n (%) = 49.3%	SMS+PS (n = 118) n (%) = 10.7%	Total (n = 1098)
**Age** **18–25 y** **26–40 y** **>40 y**	116 (26.4) 168 (38.3) 155 (35.3)	226 (41.8) 180 (33.3) 135 (25.0)	59 (50.0) 28 (23.7) 31 (26.3)	401 (36.5) 376 (34.2) 321 (29.2)
**Gender** **Male** **Female** **Other Gender**	201 (45.8) 225 (51.3) 13 (3.0)	227 (42.0) 301 (55.6) 13 (2.4)	40 (33.9) 72 (61.0) 6 (5.1)	468 (42.6) 598 (54.5) 32 (2.9)
**Ethnicity** **Caucasian** **Indigenous** **Black people** **Asian** **Mixed/Other**	281 (64.0) 44 (10.0) 44 (10.0) 45 (10.3) 25 (5.7)	323 (59.7) 48 (8.9) 63 (11.6) 66 (12.2) 41 (7.6)	71(60.2) 10 (8.5) 13 (11.0) 14 (11.9) 10 (8.5)	675 (61.5) 102 (9.3) 120 (10.9) 125 (11.4) 76 (6.9)
**Education Level** **Less than High School** **High School Diploma** **Post-secondary Education** **Prefer not to say**	22 (5.0) 211 (48.1) 197 (44.9) 9 (2.1)	15 (2.8) 286 (52.9) 221 (40.9) 19 (3.5)	4 (3.4) 68 (57.6) 40 (33.9) 6 (5.1)	41 (3.7) 565 (51.5) 458 (41.7) 34 (3.1)
**Relationship Status** **Married/Partnered/Common-Law** **Single** **Separated or Divorced** **Widowed** **Prefer not to say**	103 (29.6) 256 (58.3) 39 (8.9) 6 (1.4) 8 (1.8)	160 (29.6) 323 (59.7) 39 (7.2) 3 (0.6) 16 (3.0)	31 (26.3) 68 (57.6) 8 (6.8) 3 (2.5) 8 (6.8)	321 (29.2) 647 (58.9) 86 (7.8) 12 (1.1) 32 (2.9)
**Employment status** **Employed** **Unemployed** **Student** **Retired** **Other**	139 (31.7) 236 (53.8) 21 (4.8) 34 (7.7) 9 (2.1)	156 (28.8) 283 (52.3) 56 (10.4) 26 (4.8) 20 (3.7)	31 (26.3) 62 (52.5) 12 (10.2) 6 (5.1) 7 (5.9)	326 (29.7) 585 (52.9) 89 (8.1) 68 (6.0) 36 (3.3)
**Housing Status** **Own Home** **Rented Accommodation** **Live with Family or Friend** **Couchsurfing/Shelter/Street/Other**	105 (23.9) 148 (33.7) 159 (36.2) 27 (6.2)	98 (18.1) 169 (31.3) 238 (44.1) 35 (6.5)	15 (12.7) 36 (30.5) 61 (51.7) 6 (5.1)	218 (19.9) 355 (32.2) 458 (41.8) 68 (6.2)
**Primary Mental Health Diagnosis** **Depression** **Bipolar Disorder** **Anxiety** **Schizophrenia** **Personality Disorder** **Substance Use Disorder** **Other**	129 (29.4) 74 (16.9) 55 (12.5) 71 (16.2) 28 (6.4) 10 (2.3) 72 (16.4)	140 (25.9) 126 (23.3) 65 (12.0) 88 (16.3) 53 (9.8) 5 (0.9) 63(11.7)	21 (17.8) 23 (19.5) 21 (17.8) 19 (16.1) 24 (20.3) 0 (0.0) 10 (8.5)	290 (26.4) 223 (20.3) 141 (12.9) 178 (16.2) 105 (9.6) 15 (1.4) 145 (13.2)

**Table 3:** Descriptive mean scores of outcome measures and ANCOVA test parameters for the TAU, SMS and SMS+PS groups.

Measures	Descriptives			ANCOVA Parameters																	
	Baseline Mean (SD)			6-Weeks Mean (SE)						3-Months Mean (SE)						6-Months Mean (SE)					
	TAU	SMS	SMS+PS	TAU	SMS	SMS+PS	F Value (df)	*p*-Value	Partial eta	TAU	SMS	SMS+PS	F Value (df)	*p*-Value	Partial eta	TAU	SMS	SMS+PS	F Value (df)	*p*-Value	Partial eta
**GAD-7**	8.78 (5.76)	8.32 (5.71)	7.77 (4.67)	9.05 (0.68)	8.65 (0.66)	8.54 (1.16)	0.12	0.89	0.00	10.09 (0.76)	9.17 (1.19)	9.58 (1.53)	0.23	0.80	0.00	8.24 (0.88)	7.58 (0.74)	9.83 (1.58)	2.78	0.01 *	0.10
**PHQ-9**	11.01 (6.56)	11.39 (6.80)	11.26 (5.27)	12.51 (0.77)	11.07 (0.75)	11.61 (1.24)	0.91	0.40	0.01	12.84 (0.96)	11.75 (1.22)	11.97 (1.94)	0.28	0.76	0.00	11.42 (1.11)	11.08 (0.95)	11.64 (1.73)	2.50	0.03 *	0.09
**WHO-5**	51.68 (23.38)	52.02 (23.43)	47.80 (21.33)	46.88 (2.50)	49.48 (2.40)	44.78 (4.02)	4.24	0.00	0.10	48.24 (3.00)	47.24 (3.82)	42.07 (6.12)	1.89	0.09	0.06	47.95 (3.46)	50.31 (2.95)	48.78 (5.39)	0.14	0.87	0.00
**Suicidal Ideation**	0.76 (0.96)	0.79 (0.99)	0.75 (0.81)	0.93 (0.11)	0.74 (0.11)	0.65 (0.18)	1.15	0.32	0.01	1.11 (0.31)	1.00 (0.40)	0.75 (0.63)	0.14	0.87	0.00	0.79 (0.14)	0.82 (0.12)	1.25 (0.22)	2.00	0.07	0.07
**Sleep Issues**	1.44 (1.06)	1.48 (1.04)	1.49 (0.86)	1.55 (0.13)	1.47 (0.13)	1.61 (0.21)	0.18	0.84	0.00	1.80 (0.76)	2.09 (0.96)	1.52 (1.53)	0.06	0.094	0.00	1.66 (0.21)	1.61 (0.18)	1.66 (0.33)	0.72	0.63	0.03

* Significant *p*-value.

**Table 4:** Pairwise comparison of the 6-month mean scores on the GAD-7, PHQ-9 and WHO-5 scales for participants of TAU, SMS and SMS+PS groups.

Measures	Study Groups	Paired Groups	Mean Difference	S.E	*p*-Value	95% Confidence Interval for Difference	
						Lower Bound	Upper Bound
**GAD-7**	TAU	SMS	0.67	1.51	0.56	−1.61	2.94
		PS	−1.59	1.81	0.38	−5.18	2.00
	SMS	TAU	−0.67	1.51	0.56	−2.94	1.61
		PS	−2.26	1.75	0.20	−5.71	1.20
	PS	TAU	1.59	1.81	0.38	−2.00	5.18
		SMS	2.26	1.75	0.20	−1.20	5.71
**PHQ-9**	TAU	SMS	0.35	1.46	0.81	−4.27	3.84
		PS	−0.21	2.05	0.92	−4.27	3.84
	SMS	TAU	−0.35	2.46	0.81	−3.23	2.53
		PS	−0.56	1.97	0.78	−4.44	3.33
	PS	TAU	0.21	2.05	0.92	−3.84	4.27
		SMS	0.56	1.97	0.78	−3.33	4.44
**WHO-5**	TAU	SMS	−2.33	4.55	0.61	−11.31	6.65
		PS	−0.80	6.41	0.90	−13.46	11.86
	SMS	TAU	2.33	4.55	0.61	−6.65	11.31
		PS	1.53	6.14	0.80	−10.60	13.65
	PS	TAU	0.80	6.41	0.90	−11.86	13.46
		SMS	−1.53	6.14	0.80	−13.65	10.60

## Data Availability

The datasets used and/or analysed during the current study are available from the corresponding author upon reasonable request.

## Abbreviations

The following abbreviations are used in this manuscript:

ED	Emergency Department
CIHI	Canadian Institute for Health Information
TAU	Treatment as Usual
SMS	Supportive Text Messaging
SMS+PS	Supportive Text Messaging Plus Peer Support
ORs	Odds Ratios
CIs	Confidence Intervals
GAD-7	Generalized Anxiety Disorder Scale
PHQ-9	Patient Health Questionnaire
WHO-5	World Health Organization-Five Well-Being Index
ANCOVA	Analysis of Covariance

## References

[1] Mao W., Shalaby R., Owusu E., Elgendy H., Shalaby N., Agyapong B., Nichols A., Eboreime E., Nkire N., Agyapong V.I.O. (2023). Status after Hospital Discharge: An Observational Study of the Progression of Patients’ Mental Health Symptoms Six Weeks after Hospital Discharge. J. Clin. Med..

[2] Smith T.E., Haselden M., Corbeil T., Wall M.M., Tang F., Essock S.M., Frimpong E., Goldman M.L., Mascayano F., Radigan M. (2020). Effect of scheduling a post-discharge outpatient mental health appointment on the likelihood of successful transition from hospital to community-based care. J. Clin. Psychiatry.

[3] Gillard S., White R., Miller S., Turner K. (2015). Open access support groups for people experiencing personality disorders: Do group members’ experiences reflect the theoretical foundations of the SUN project?. Psychol. Psychother. Theory Res. Pract..

[4] Vigod S.N., Kurdyak P.A., Dennis C.-L., Leszcz T., Taylor V.H., Blumberger D.M., Seitz D.P. (2013). Transitional interventions to reduce early psychiatric readmissions in adults: Systematic review. Br. J. Psychiatry.

[5] Canadian Institute for Health Information (2008). Health Indicators. https://secure.cihi.ca/free_products/HealthIndicators2008_FRweb.pdf.

[6] Eboreime E., Shalaby R., Mao W., Owusu E., Vuong W., Surood S., Bales K., MacMaster F.P., McNeil D., Rittenbach K. (2022). Reducing readmission rates for individuals discharged from acute psychiatric care in Alberta using peer and text message support: Protocol for an innovative supportive program. BMC Health Serv. Res..

[7] Herman D., Conover S., Gorroochurn P., Hinterland K., Hoepner L., Susser E. (2011). A randomized trial of critical time intervention in persons with severe mental illness following institutional discharge. Psychiatr. Serv..

[8] Forchuk C., Martin M.L., Jensen E., Ouseley S., Sealy P., Beal G., Reynolds W., Sharkey S. (2013). Integrating an evidence-based intervention into clinical practice: ‘Transitional relationship model’. J. Psychiatr. Ment. Health Nurs..

[9] Wakeman S.E., Rigotti N.A., Chang Y., Herman G.E., Erwin A., Regan S., Metlay J.P. (2019). Effect of integrating substance use disorder treatment into primary care on inpatient and emergency department utilization. J. Gen. Intern. Med..

[10] Abraham O., Myers M.N., Brothers A.L., Montgomery J., Norman B.A., Fabian T. (2017). Assessing need for pharmacist involvement to improve care coordination for patients on LAI antipsychotics transitioning from hospital to home: A work system approach. Res. Soc. Adm. Pharm..

[11] Shaw H., Mackie C.A., Sharkie I. (2000). Evaluation of effect of pharmacy discharge planning on medication problems experienced by discharged acute admission mental health patients. Int. J. Pharm. Pract..

[12] Attfield J., Brown S., Carter T., Callaghan P. (2017). A retrospective case comparison study of the relationship between an Integrated Care Pathway for people diagnosed with schizophrenia in acute mental health care and service users’ length of stay, readmission rates and follow-up within 7 days of discharge. J. Psychiatr. Ment. Health Nurs..

[13] Harris P.A., Taylor R., Thielke R., Payne J., Gonzalez N., Conde J.G. (2009). Research electronic data capture (REDCap)—A metadata-driven methodology and workflow process for providing translational research informatics support. J. Biomed. Inform..

[14] (2024). calculator.net. Sample Size Calculator. https://www.calculator.net/sample-size-calculator.html?type=1&cl=95&ci=3&pp=50&ps=4756408&x=Calculate.

[15] Williams N. (2014). The GAD-7 questionnaire. Occup. Med..

[16] Kroenke K., Spitzer R.L., Williams J.B., Monahan P.O., Löwe B. (2007). Anxiety disorders in primary care: Prevalence, impairment, comorbidity, and detection. Ann. Intern. Med..

[17] Kroenke K., Spitzer R.L., Williams J.B. (2001). The PHQ-9: Validity of a brief depression severity measure. J. Gen. Intern. Med..

[18] Mao W., Adu M., Eboreime E., Shalaby R., Nkire N., Agyapong B., Pazderka H., Obuobi-Donkor G., Owusu E., Oluwasina F. (2022). Post-traumatic stress disorder, major depressive disorder, and wildfires: A fifth-year postdisaster evaluation among residents of Fort McMurray. Int. J. Environ. Res. Public Health.

[19] Beard C., Hsu K., Rifkin L., Busch A., Björgvinsson T. (2016). Validation of the PHQ-9 in a psychiatric sample. J. Affect. Disord..

[20] Lequerica A.H., Watson E., Dijkers M.P., Goldin Y., Hoffman J.M., Niemeier J.P., Silva M.A., Rabinowitz A., Chiaravalloti N.D. (2022). The utility of the Patient Health Questionnaire (PHQ-9) sleep disturbance item as a screener for insomnia in individuals with moderate to severe traumatic brain injury. J. Head Trauma Rehabil..

[21] Morin C.M. (1993). Insomnia: Psychological Assessment and Management.

[22] Simon G.E., Rutter C.M., Peterson D., Oliver M., Whiteside U., Operskalski B., Ludman E.J. (2013). Do PHQ depression questionnaires completed during outpatient visits predict subsequent suicide attempt or suicide death?. Psychiatr. Serv..

[23] MacGregor K.L., Funderburk J.S., Pigeon W., Maisto S.A. (2012). Evaluation of the PHQ-9 Item 3 as a screen for sleep disturbance in primary care. J. Gen. Intern. Med..

[24] Louzon S.A., Bossarte R., McCarthy J.F., Katz I.R. (2016). Does suicidal ideation as measured by the PHQ-9 predict suicide among VA patients?. Psychiatr. Serv..

[25] Bauer A.M., Chan Y.-F., Huang H., Vannoy S., Unützer J. (2013). Characteristics, management, and depression outcomes of primary care patients who endorse thoughts of death or suicide on the PHQ-9. J. Gen. Intern. Med..

[26] Topp C.W., Østergaard S.D., Søndergaard S., Bech P. (2015). The WHO-5 Well-Being Index: A systematic review of the literature. Psychother. Psychosom..

[27] De Wit M., Pouwer F., Gemke R.J., Delemarre-Van De Waal H.A., Snoek F.J. (2007). Validation of the WHO-5 Well-Being Index in adolescents with type 1 diabetes. Diabetes Care.

[28] IBM Corp (2017). IBM SPSS Statistics for Mac.

[29] Chung D.T., Ryan C.J., Hadzi-Pavlovic D., Singh S.P., Stanton C., Large M.M. (2017). Suicide rates after discharge from psychiatric facilities: A systematic review and meta-analysis. JAMA Psychiatry.

[30] Walter F., Carr M.J., Mok P.L., Antonsen S., Pedersen C.B., Appleby L., Fazel S., Shaw J., Webb R.T. (2019). Multiple adverse outcomes following first discharge from inpatient psychiatric care: A national cohort study. Lancet Psychiatry.

[31] Olfson M., Marcus S.C., Doshi J.A. (2010). Continuity of care after inpatient discharge of patients with schizophrenia in the Medicaid program: A retrospective longitudinal cohort analysis. J. Clin. Psychiatry.

[32] Agyapong V.I., Mrklas K., Juhás M., Omeje J., Ohinmaa A., Dursun S.M., Greenshaw A.J. (2016). Cross-sectional survey evaluating Text4Mood: Mobile health program to reduce psychological treatment gap in mental healthcare in Alberta through daily supportive text messages. BMC Psychiatry.

[33] Tyler N., Wright N., Waring J. (2019). Interventions to improve discharge from acute adult mental health inpatient care to the community: Systematic review and narrative synthesis. BMC Health Serv. Res..

[34] Meng N., Liu R., Wong M., Liao J., Feng C., Li X. (2020). The association between patient-reported readiness for hospital discharge and outcomes in patients diagnosed with anxiety disorders: A prospective and observational study. J. Psychiatr. Ment. Health Nurs..

[35] Newnham E.A., Hooke G.R., Page A.C. (2010). Progress monitoring and feedback in psychiatric care reduces depressive symptoms. J. Affect. Disord..

[36] Puschner B., Steffen S., Völker K., Spitzer C., Gaebel W., Janssen B., Klein H.E., Spiessl H., Steinert T., Grempler J. (2011). Needs-oriented discharge planning for high utilisers of psychiatric services: Multicentre randomised controlled trial. Epidemiol. Psychiatr. Sci..

[37] Balaban R.B., Weissman J.S., Samuel P.A., Woolhandler S. (2008). Redefining and redesigning hospital discharge to enhance patient care: A randomized controlled study. J. Gen. Intern. Med..

[38] Marissa L. (2022). Evidence-Based Best Practice for Discharge Planning: A Policy Review. https://soar.usa.edu/scholprojects/79/.

[39] Kuwabara A., Su S., Krauss J. (2020). Utilizing digital health technologies for patient education in lifestyle medicine. Am. J. Lifestyle Med..

[40] Albarqi M.N. (2024). Exploring the effectiveness of technology-assisted interventions for promoting independence in elderly patients: A systematic review. Healthcare.

[41] Moggia D., Lutz W., Brakemeier E.L., Bickman L. (2024). Treatment Personalization and Precision Mental Health Care: Where are we and where do we want to go?. Adm. Policy Ment. Health Ment. Health Serv. Res..

[42] Isaacs A.N., Mitchell E.K.L. (2024). Mental health integrated care models in primary care and factors that contribute to their effective implementation: A scoping review. Int. J. Ment. Health Syst..

[43] Ojo S., Okoye T.O., Olaniyi S.A., Ofochukwu V.C., Obi M.O., Nwokolo A.S., Okeke-Moffatt Chinwe Iyun Oluwatosin B., Idemudia Etinosa A., Obodo Okiemute R. (2024). Ensuring continuity of care: Effective strategies for the post-hospitalization transition of psychiatric patients in a family medicine outpatient clinic. Cureus.

